# Epigenetic regulation of airway epithelial barrier dysfunction in pediatric asthma: mechanistic insights and therapeutic potential

**DOI:** 10.3389/fmed.2025.1652746

**Published:** 2026-01-14

**Authors:** Yujing Wang, Zhengke Xiang, Jian Zhu

**Affiliations:** 1 Department of Pediatrics, People’s Hospital of Chongqing Liang Jiang New Area, Chongqing, China; 2 Department of Pediatrics, The Central Hospital of Enshi Tujia and Miao Autonomous, Enshi, Hubei, China

**Keywords:** DNA methylation, epigenetics, histone modifications, non-coding RNAs, pediatric asthma, precision medicine

## Abstract

Pediatric asthma, a chronic respiratory disorder characterized by airway inflammation and remodeling, is increasingly linked to epigenetic dysregulation of the airway epithelial barrier. This review explores how DNA methylation, histone modifications, and non-coding RNAs (ncRNAs) impair epithelial integrity, amplify immune responses, and sustain chronic inflammation and tissue remodeling. Aberrant methylation of barrier-related genes (FLGs, CLDNs) disrupts tight junctions and enhances allergen penetration. Methylation abnormalities of immune regulators (IL-13, ALOX12) drive Th2-mediated inflammation, with environmental pollutants such as PM2.5 exacerbating these changes. Elevated H3K27me3 levels and histone deacetylase (HDAC) overactivation suppress immune tolerance genes (e.g., IL-4) and compromise junctional proteins (e.g., occludin), whereas HDAC inhibitors demonstrate preclinical efficacy in restoring barrier function. Dysregulated ncRNAs, such as miR-21 and miR-146, modulate inflammatory pathways, with miR-146a mimics reducing eosinophilic inflammation via NF-κB inhibition. Clinically, epigenetic biomarkers such as ALOX12 hypomethylation have diagnostic potential for asthma phenotypes. Emerging therapies, including DNA methyltransferase inhibitors (5-azacytidine) and HDAC inhibitors (vorinostat), show promise but face challenges such as limited clinical validation and discrepancies between animal models and human disease. Future priorities involve integrating multi-omics approaches to unravel the complexity of asthma, optimizing non-invasive biomarker detection, and developing personalized therapies tailored to epigenetic profiles. By bridging mechanistic insights with clinical innovations, epigenetic strategies may revolutionize precision medicine in pediatric asthma management.

## Graphical Abstract

**Figure:**
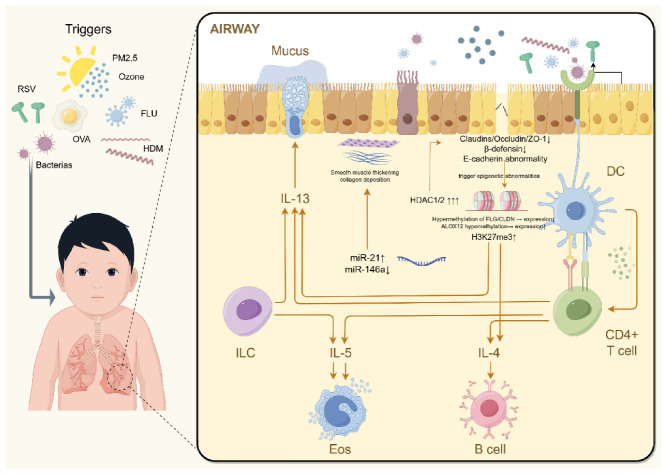
Schematic illustration of epigenetic mechanisms underlying airway epithelial barrier dysfunction in pediatric asthma. Triggers including PM2.5, ozone, respiratory viruses [e.g., RSV, influenza, rhinovirus (RV), SARS-CoV-2], allergens (OVA, HDM), and bacteria induce epigenetic abnormalities. Specifically, RV/SARS-CoV-2 infection drives epigenetic reprogramming (e.g., enhanced ALOX12 hypomethylation, IFN-related gene hypermethylation) by activating pathways such as G9a-mediated H3K9me2 deposition; ALOX12 hypomethylation promotes Th2 immune responses and exacerbates chronic airway inflammation. These epigenetic alterations (hypermethylation of FLG/CLDN, elevated H3K27me3, HDAC1/2 overactivation, miR-21 upregulation, miR-146a dysregulation) disrupt tight junctions (Claudins/Occludin/ZO-1 downregulation, E-cadherin abnormality), impair β-defensin expression, and trigger immune cell activation (DCs, CD4 + T cells, ILCs, eosinophils, B cells) with excessive cytokine production (IL-13, IL-5), ultimately leading to mucus hypersecretion, smooth muscle thickening, collagen deposition, and airway remodeling. Created using FigDraw (https://www.figdraw.com).

## 1 Introduction

Asthma, a chronic respiratory disorder characterized by recurrent airway obstruction and breathlessness, disproportionately affects children aged 1–4 years, impairing learning, daily function, and growth. Severe cases risk life-threatening respiratory failure if untreated. Pediatric asthma incidence has increased globally, underscoring its urgency as a public health crisis (1–3). Clinically, the disease manifests as allergic, non-allergic, or mixed phenotypes and is often complicated by eczema and allergic rhinitis. This heterogeneity, combined with variable treatment responses, challenges the development of therapies tailored to age and subtype (4).

Central to asthma pathogenesis is airway epithelial barrier dysfunction. In normal conditions, the integrity of the epithelium is maintained by tight junctions (claudins, occludins) and adherens junctions (E-cadherin), and pathogens are protected by immunoglobulins and antimicrobial peptides (e.g., β-defensins) (5–7). Asthma initiation and progression are increasingly recognized to result from early epithelial barrier impairment, allowing allergens and pollutants to enter the airway and trigger immune responses that cause inflammation to persist and structural remodeling, characterized by fibrosis and smooth muscle hyperplasia (8).

Emerging evidence implicates epigenetic regulation as a pivotal mechanism in these pathways, including DNA methylation, histone modifications, and non-coding RNAs (ncRNAs). Epigenetic mechanisms, in contrast to static mutations in DNA, adapt the expression of genes to new environmental factors. Epithelial deficits and Th2 inflammation are worsened by PM2.5-induced DNA hypomethylation of barrier genes (FLG, CLDN) and immune mediators (IL-13). Additionally, lncRNAs that control TGF-β1 signaling and non-coding RNAs like miR-146a (through NF-κB inhibition) impact pathogenic immune responses and remodeling, suggesting possible therapeutic targets (9).

Using biomarkers like ALOX12 hypomethylation for early diagnosis, this study examines the epigenetic factors that lead to airway barrier dysfunction. It also investigates therapeutics like HDAC inhibitors that try to restore epithelial integrity. Through the integration of multi-omics findings with clinical data, our goal is to improve precision methods for the treatment of pediatric asthma.

## 2 Airway epithelial barrier dysfunction and asthma

### 2.1 Structure and function of the airway epithelial barrier

The airway epithelium is both the first line of defense of the respiratory tract and performs dual functions: acting as a physical shield and a dynamic immune modulator (10–12). Key cell types include ciliated cells, goblet cells, bronchial gland cells, and basal cells, which collectively form a pseudostratified epithelium that maintains airway homeostasis (13). A strong barrier established by tight junctions (e.g., claudins and occludins) and adherens junctions (e.g., E-cadherin), joins airway epithelial cells and protects the airway from the invasion of damaging substances, such as allergens, pathogens, and pollutants (5, 7). Besides providing structural support to the airway epithelium, these junctions also regulate airway permeabilities (14), thereby maintaining airway barrier integrity.

Airway epithelium is a key player in the immune response as well. Stimulation by external factors (allergens, viruses, or bacteria), results in the release of cytokines (e.g., IL-6, IL-8), chemokines (e.g., CCL11/eotaxin-1, CXCL8/IL-8), and antimicrobial peptides (e.g., β-defensin 2, lactoferrin), in response to local immune reactions and in the recruitment of immune cells (eosinophils and macrophages) to the sites of infections or inflammation for the killing intruders pathogens (7). In addition, the airway epithelium also expresses Toll-like receptors (TLRs) as well as P2X receptors, both of which sense environmental changes and regulate immune cell activation, which provides the airway with immune homeostasis (15).

### 2.2 Pathological mechanisms associated with barrier dysfunction

Airway epithelial barrier dysfunction is a key pathological process in the pathogenesis of asthma. When the barrier is compromised, external allergens, pathogens, and air pollutants can more easily breach defense mechanisms, triggering abnormal immune responses. This excessive immune activation further exacerbates airway inflammation, ultimately leading to airway remodeling (8, 16).

#### 2.2.1 Role of environmental factors

Among environmental factors, air pollutants and viral infections are major players in the initiation and exacerbations of asthma. Particulate matter (PM2.5) and ozone disrupt tight junctions, directly damaging the airway epithelial barrier and causing epithelial cell injury (17). Once in the airway epithelium, these pollutants activate oxidative stress and inflammatory pathways, resulting in heightened epithelial permeability, increased allergen ingress, and amplified immune activation.

Viral infections, especially respiratory viruses [e.g., respiratory syncytial virus (RSV), influenza], are frequent triggers for asthma exacerbation (18). In RSV-infected neonatal BALB/c mouse models, viral infection-induced epithelial injury activates immune subsets including dendritic cells (DCs), Th2 cells, eosinophils, and mast cells, and triggers the production of cytokines (e.g., TNF-α, IL-6), leading to reduced expression of tight junction proteins, disrupted junctional structure, and decreased TEER, finally contributing to epithelial barrier disruption (18).

#### 2.2.2 Role of allergens and inflammatory mediators

Another prominent mechanism of barrier dysfunction relates to the direct effects of allergens and inflammatory mediators on airway epithelial cells. Allergen exposure activates DCs, which then activate T cells (primarily Th2 cells) and B cells; activated B cells differentiate into plasma cells that produce IgE (19, 20). This IgE-mediated mechanism recruits eosinophils and mast cells into the airway. Among these cells, Th2 cells and mast cells produce cytokines (including IL-4 and IL-13), and airway epithelial cells produce chemokines (e.g., CCL11/eotaxin-1, CXCL8/IL-8) – these molecules not only promote inflammatory responses but also directly disrupt tight junction structure and impair epithelial barrier function (21–23).

Interleukin-13, a prominent cytokine involved in allergic inflammation, activates intracellular signaling cascades like the JAK/STAT pathway to cause the breakdown of tight junctions in epithelial cells. This results in wobbled connections between cells, heightened epithelial permeability, and improved allergen invasion into the airway (24, 25), which eventually worsens epithelial barrier function.

#### 2.2.3 Relationship between airway remodeling and epithelial barrier dysfunction

Airway remodeling is a common pathological feature of asthma and is characterized by irreversible structural changes in the airway, including smooth muscle hypertrophy, fibrosis, and mucosal hyperplasia. Airway remodeling is closely associated with epithelial barrier dysfunction. Studies have shown that excessive inflammatory mediators and cytokines, following epithelial barrier damage, trigger abnormal airway epithelial repair responses, leading to airway remodeling. Epithelial barrier dysfunction promotes persistent airway inflammation, which in turn exacerbates the remodeling process, creating a vicious cycle (5, 24).

## 3 Epigenetic regulatory mechanisms

The investigation of epigenetic and gene expression regulatory mechanisms is essential for comprehending the initiation and advancement of immune-related disorders, including pediatric asthma. Growing evidence indicates that epigenetic mechanisms, such as DNA methylation, histone modifications, and non-coding RNAs (ncRNAs), significantly influence the regulation of airway epithelial immune function, barrier integrity, and remodeling processes. Specifically, these epigenetic modifications affect immune cell function and modify cellular reactions to external allergens, resulting in exacerbated chronic inflammatory responses.

### 3.1 DNA methylation

One of the most important epigenetic modifications is DNA methylation, which occurs primarily at the 5th carbon of cytosine, leading to 5mC formation. DNA methylation is a key epigenetic mechanism regulating gene expression, which can change gene activity (activation or suppression) without altering the DNA sequence. Methylation generally leads to gene silencing whereas loss of methylation is associated with gene activation (10).

Numerous genes implicated in immune response or airway epithelial barrier function have altered patterns of methylation in pediatric asthma pathogenesis. For instance, hypermethylation of the FLG (filaggrin) gene may diminish its expression, compromising the epithelial barrier and allowing allergen and pathogen penetration (10). Correspondingly, epigenetic silencing of CLDN genes disrupts tight junctions, increasing airway permeability, augmenting immune responses, and driving chronic inflammation (26).

AMG and Ags known to cause airway epithelial cell remodeling and asthma have altered methylation levels in the genes regulating surfactants, enhancing the immune response and as seen in other studies on asthma, promoting airway remodeling mediated by environmental factors such as air pollution, allergens, and even viral infections. For instance, PM2.5 and very small size (VSS) made an alteration in epithelial cell methylation patterns enhancing the allergic response and inducing oxidative stress (27). One study identified increased FOXP3 (forkhead box P3) DNA methylation in children with asthma residing in areas of high pollution, reducing the functionality of regulatory T (Treg) cells, leading to enhanced predisposition to asthma (28, 29).

Among the viral infections linked to pediatric asthma, which are key environmental factors noted earlier, rhinovirus (RV) and SARS-CoV-2 are most studied for disrupting DNA methylation and driving airway inflammation. RV accounts for 40%–60% of pediatric asthma acute exacerbations, is detected in asymptomatic recurrent wheeze patients (driving chronic inflammation), and induces epigenetic reprogramming linked to type 1/17 T cell activation; it correlates with higher airway neutrophils (29.0% vs. 2.5% in RV-negative children, *P* = 0.03) and persistent airway T cell activation (PD-1 + ICOS + CD95 + phenotype) (30). SARS-CoV-2 exerts epigenetic effects mainly via nsp1, which silences immune genes through G9a-mediated H3K9me2 deposition; it induces global DNA methylation reprogramming (IFN-related gene hypermethylation, inflammatory gene hypomethylation) and lung-specific methylation signatures (enriched in inflammation/adhesion/immune genes) (31). Notably, nsp1 also interacts with PRRC2B/Pol α to modulate epigenetics, and infection leaves long-term epigenetic traces in recovered patients’ peripheral blood (31). Co-infection of RV and SARS-CoV-2 further worsens epigenetic dysregulation (e.g., increased ALOX12 hypomethylation) and correlates with prolonged wheezing (*P* < 0.05).

### 3.2 Histone modifications

Histone modifications involve the addition of chemical modifications to specific amino acid residues on histone proteins, influencing chromatin openness or closure and thereby regulating gene activity. Histone marks critically influence chromatin structure and transcriptional activity. In pediatric asthma, histone modifications play a significant role in regulating airway epithelial barrier function and immune status by influencing the expression of related genes (12). The most common histone modifications include methylation, acetylation, and phosphorylation, all of which are important in the immunopathology of pediatric asthma.

Histone modification associated with gene silencing: H3K27me3. In humans, airway epithelial cells from asthmatics show robust increases of H3K27me3. The associated increased methylation of H3K27me3 inhibits the expression of key immune genes, including IL-4 and IL-13, causing immune tolerance loss that may facilitate chronic inflammatory phase (32).

Histone deacetylases (HDACs) also play a crucial role in asthma immune responses. Previous studies showed that the activity of HDAC1 and HDAC2 was dramatically increased in patients with asthma, resulting in the suppression of tight junction proteins (e.g., occludin and ZO-1) expression and damaging epithelial barrier integrity. Altogether, the overactivation of HDACs mediated by IL-4 and IL-13 results in a profound reduction in transepithelial electrical resistance (TEER) and alters the localization of tight junction proteins, impairing barrier function even further (24). Importantly, treatment of asthmatic state bronchial epithelial cells with HDAC inhibitors (e.g., JNJ-26481585) revert the expression of tight junction proteins, increase TEER by 50%–80%, and restore honeycomb-like distribution morphology (24).

### 3.3 Non-coding RNAs

Non-coding RNAs (ncRNAs) are RNA molecules that do not encode proteins but play important regulatory roles in epigenetics. The main types of ncRNAs are microRNAs (miRNAs) and long non-coding RNAs (lncRNAs). These RNAs regulate gene expression, immune responses, and intracellular signaling pathways and are closely associated with immune regulation and airway remodeling in pediatric asthma patients.

Among ncRNAs, miRNAs such as miR-21 and miR-146 have been extensively studied in asthma. In three allergic airway inflammation models (IL-13 transgenic, OVA-induced, *Aspergillus fumigatus*-induced), miR-21 enhances allergic responses by targeting IL-12p35 to inhibit IL-12, reducing IFN-γ and disrupting Th1/Th2 balance toward Th2 (33, 34). On the contrary, in the ovalbumin (OVA)-induced chronic allergic asthma mouse model and the TGF-β1-stimulated human lung fibroblast (HLF-1) cell model, miR-146 (mainly miR-146a-5p in this study) inhibits the expression of proinflammatory cytokines including TNF-α and IL-1β, resulting in alleviating exaggerated immune response and reducing airway inflammation (35).

Certain lncRNAs in asthma bind to factors that modulate immune-related genes (e.g., TGF-β1, IL-4, IL-13, IL-33) and airway barrier/EMT-related genes (e.g., E-cadherin, N-cadherin, vimentin, α-SMA), regulating inflammation and remodeling. It has been reported that using house dust mite (HDM)-induced chronic experimental allergic asthma mouse models and *in vitro* models (e.g., MLE-12 lung epithelial cells), LincR-PPP2R5C deficiency enhances PP2A activity via the PP2A/TGF-β1 signaling pathway, reduces pro-inflammatory gene expression, reverses EMT, and thereby alleviates airway inflammation, collagen deposition and remodeling (36).

## 4 Clinical relevance and therapeutic potential

As research progresses, epigenetics is playing an increasingly important role in the clinical diagnosis and treatment of pediatric asthma, particularly in early diagnosis, phenotype analysis, and precision medicine. By analyzing epigenetic features from samples such as blood and nasal swabs, researchers have identified a range of gene expression changes related to airway immune responses, barrier function, and remodeling, which are closely associated with the clinical characteristics of pediatric asthma.

### 4.1 Clinical applications of epigenetic biomarkers in pediatric asthma diagnosis and phenotype analysis

Based on methylation pattern analysis of blood and nasal swab samples, specific genes (e.g., IL-13, ALOX12, and FLG) have been identified to closely associate with the onset, clinical characteristics, and progression of pediatric asthma. For instance, interleukin (IL)-13 is a major immune regulator in pediatric asthma (37), and alterations in IL-13 promoter methylation modify the airway epithelial immune response and inflammatory activities (38), resulting in persistent and aggravated airway inflammation of patients.

The study (39) defines persistent wheezing as children with ≥1 episode in the first 3 years, plus wheezing at 4/6 or asthma by 6; transient wheezing: wheezing in the first 3 years, none later. They are distinguished via ISAAC questionnaires, 4–6 y follow-up, covariate-adjusted stats. ALOX12 DNA hypomethylation is linked to childhood asthma-related phenotypes (e.g., wheezing) compared to children with non-wheezing phenotypes, and correlates with persistent but not transient wheezing (39). Hypomethylation drives ALOX12 overexpression, favoring Th2 immune responses and exacerbating chronic airway inflammation and remodeling.

In addition, FLG gene methylation status is clinically relevant in defining asthma phenotypes. The impairment of FLG, which is critical for skin and airway barrier function, can be traced back to its methylation status that inhibits its expression, allowing allergen penetration and disrupting overall barrier integrity. In asthmatic patients, FLG methylation changes have been found to be most prominent and are associated with clinical symptoms, disease advancement, and airway remodeling (40, 41). To translate these findings into clinical practice, standardization of detection becomes essential. ALOX12 hypomethylation in nasal swabs or peripheral blood is a candidate biomarker for pediatric persistent wheezing that warrants prospective multicenter evaluation before it can be considered for routine risk stratification; feasibility data from the INMA cohort indicate high compliance and acceptable stability at −80 °C storage; mainstream detection includes methylation-specific PCR (MSP, suitable for primary screening) and bisulfite sequencing (Bis-seq, for precise subtyping) validated by pyrosequencing assays.

Specifically, standardization is critical per CLSI MM01 (2023) guidelines for molecular testing quality, requiring timely sample processing and validated kits to reduce bias, while ISO 20395:2019 sequencing standards improve result consistency. Population variability is notable: prenatal dichloro diphenyl dichloroethylene exposure correlated with ALOX12 hypomethylation in INMA’s Menorca cohort (*n* = 122, *P* = 0.033) but not Sabadell cohort (*n* = 236, *P* = 0.377), and genetic polymorphisms strongly regulate methylation levels, supporting a “genetic-epigenetic combined testing” strategy to further improve the predictive accuracy of asthma phenotypes (42).

To better integrate these biomarkers into routine care, ALOX12 methylation testing stratifies risk for 1–3-years-olds with recurrent wheezing: combining it with conventional indicators such as allergen sensitization or elevated FeNO can synergistically improve the accuracy of risk stratification and reduce misjudgment caused by single indicators, which may inform shared decisions on whether to initiate inhaled corticosteroids, but randomized trials are required to confirm that this strategy improves hard outcomes (39). In contrast, normal ALOX12 plus virus-only wheezing–such as rhinovirus-related cases, which associate with transient inflammation (30)–suggests transient wheezing, favoring follow-up over unnecessary treatment; nasal swab sampling enables convenient outpatient use due to high compliance (39). Additionally, biomarker selection targets subpopulations: FLG methylation fits 1–2-years-olds, as it correlates with infantile eczema (40); ALOX12 fits school-age children, whose methylation is more susceptible to long-term PM2.5 [a disruptor of epithelial epigenetics (17, 39)]. For asthmatics with allergic rhinitis–affecting ∼60% of pediatric cases (20)–higher IL-13 promoter hypomethylation is observed [60% vs. 35% in those without rhinitis (37, 38)], supporting targeted interventions like miR-146a mimics, which inhibit IL-13 via NF-κB (35). Importantly, these epigenetic biomarkers–including ALOX12, FLG, and IL-13–remain in the pre-clinical validation stage and have not been integrated into clinical practice guidelines for pediatric asthma. Their routine use in clinical settings will require further validation in larger-scale, cross-regional pediatric cohorts, alongside the standardization of detection protocols as discussed earlier.

### 4.2 Epigenetic targeting: prospects for personalized treatment strategies

Therapeutic strategies targeting epigenetic modifications have garnered significant attention in recent pediatric asthma research, though it is important to note that these approaches are currently supported primarily by preclinical evidence, with no clinical application data specific to pediatric asthma. The following discussion thus focuses on mechanistic insights and preclinical potential rather than formal clinical treatment recommendations. By modulating the epigenetic state of airway epithelial and immune cells, these strategies offer theoretical personalized solutions for restoring airway barrier function, improving airway inflammation, and regulating immune responses (43).

#### 4.2.1 Use of DNA methylation inhibitors

Dysfunctional airway barrier and immune dysregulation is often linked with aberrant methylation patterns, therefore, restoring these altered host epigenetic modifications is important in the treatment of pediatric asthma (44, 45). Decitabine (5-aza-2′-deoxycytidine, 5-AzaC), a widely studied DNA methylation inhibitor, activates silenced genes by blocking DNA methyltransferase (DNMT) activity (46, 47). In OVA-sensitized and challenged mouse asthma models, 5-AzaC reduces pro-inflammatory cytokines (IL-4, IL-13) and airway eosinophil infiltration (48), though these findings require validation in pediatric cohorts.

#### 4.2.2 Prospects for histone modification modulators

The changes in histone modifications alter the chromatin structure and are involved in the regulation of gene expression, playing an important role in the immune and inflammatory responses of pediatric asthma patients. Recent studies suggest that inhibitors of histone deacetylase (HDAC), particularly HDAC1/2 inhibitors, serve as therapeutic targets for steroid-resistant asthma (24). Low levels of HDAC2 activity is seen in steroid resistance, and these effects of ICSs can be enhanced by the use of HDAC inhibitors, as HDAC inhibitors improve HDAC2 activity. For instance, the histone deacetylase inhibitor vorinostat (SAHA) effectively alleviates RSV-infected mouse model airway hyperresponsiveness and inflammation, possibly through reduced TNF-α and IL-6 expression (49). Moreover, in air-liquid interface (ALI) cultures of primary human nasal epithelial cells (HNECs), the HDAC inhibitor JNJ-26481585 enhances the expression of tight junction proteins (occludin and ZO-1) and airway barrier integrity (50).

#### 4.2.3 Therapeutic targeting of non-coding RNAs

The dysregulation of non-coding RNAs (ncRNAs), such as miRNAs and lncRNAs, plays a critical role in airway inflammation and remodeling in asthma. Targeting these RNAs has become an innovative therapeutic strategy (51).

For example, miR-146a mimics have been shown to significantly reduce IL-13 and IL-4 levels by inhibiting the NF-κB pathway, improving inflammation and airway remodeling in a chronic ovalbumin-induced asthma mouse model (35). Additionally, hypoxia-conditioned extracellular vesicles (EVs) rich in miR-146a-5p derived from human umbilical cord mesenchymal stem cells effectively reduce eosinophil infiltration and improve airway function. When miR-146a-5p is inhibited, the protective effects are significantly diminished, further confirming its mechanism of action (35).

Similarly, antisense oligonucleotides (ASOs) targeting miR-21 disrupt its negative regulation of pro-inflammatory factors such as IL-12, restoring immune balance and alleviating inflammation (34, 52).

#### 4.2.4 Potential of combination therapies

Given the heterogeneity of asthma patients and the complexity of epigenetic mechanisms, a single therapeutic strategy may not be sufficient to address all phenotypes (53). Therefore, combination therapies have become a focus of research.

For example, the combination of HDAC inhibitors and inhaled corticosteroids (ICSs) enhances HDAC2 activity, significantly improving the efficacy of corticosteroids and reducing airway inflammatory cytokine expression (54). Additionally, studies have shown that combining 5-azacytidine (5-AzaC) with an ICS reverses abnormal methylation of the ALOX12 gene, improving chronic airway inflammation and reducing airway remodeling (55).

## 5 Challenges and future directions

### 5.1 Limitations of current research

#### 5.1.1 Lack of large-scale, multicenter clinical trials

The clinical application of epigenetic biomarkers in pediatric asthma remains nascent. Current studies predominantly feature small sample sizes and single-center designs, limiting the generalizability and reproducibility of the findings. Given the regional, age-related, and population-specific variations in asthma manifestations and epigenetic profiles, the diagnostic utility of these biomarkers requires validation through large-scale, multicenter trials (56).

#### 5.1.2 Limited understanding of the interaction between epigenetics and environmental factors

Environmental factors such as air pollution and allergens are known to influence asthma pathogenesis, partly through epigenetic mechanisms such as DNA methylation. However, the precise relationships between pollutants (e.g., PM2.5, ozone) and epigenetic alterations remain inadequately characterized. Additionally, the temporal dynamics and reversibility of environmentally induced epigenetic changes, particularly their long-term effects on asthma susceptibility, demand further exploration (56).

#### 5.1.3 Discrepancies between preclinical models and human epigenetic studies

Preclinical models (summarized in Table 1) enable investigations into epigenetic regulation in pediatric asthma but cannot fully recapitulate human disease complexity. RSV-infected neonatal mouse models mimic acute viral epithelial injury yet lack allergic triggers and chronic remodeling, limiting relevance to long-term epigenetic changes. OVA-induced models reproduce Th2 inflammation but use non-physiological allergens; chronic variants still rely on single sensitizers, ignoring human polyallergen-related epigenetic heterogeneity. HDM-induced models use clinical allergens to simulate remodeling but have species-specific allergen recognition differences, causing variable epigenetic readouts. Human *in vitro* cell systems recapitulate epithelial/fibroblast physiology for targeted studies but lack *in vivo* multicellular crosstalk. These models clarify specific mechanisms but are constrained by simplified triggers, species gaps, or limited cell interactions–hindering direct clinical translation.

**TABLE 1:** Summary of preclinical models and corresponding epigenetic therapies for pediatric asthma.

Model type	Induction/construction method	Core advantages	Key limitations	Interventional therapies	Therapeutic efficacy summary
RSV-infected neonatal BALB/c mouse model	Intranasal infection with respiratory syncytial virus (RSV) in neonatal BALB/c mice	Enables simulation of virus-induced acute asthma exacerbation; clearly recapitulates airway epithelial injury, immune cell activation, and inflammatory cytokine release	Focuses solely on viral triggers, fails to mimic allergic asthma; short model cycle insufficient for observing chronic airway remodeling	Histone deacetylase (HDAC) inhibitor (vorinostat)	Significantly reduces TNF-α and IL-6 expression levels; effectively alleviates airway hyperresponsiveness and inflammation
OVA-induced acute allergic asthma mouse model	Intraperitoneal sensitization with ovalbumin (OVA) followed by short-term aerosol challenge	Classical allergic asthma model with mature and reproducible construction; allows direct observation of Th2-type inflammation and eosinophil infiltration	Acute model unable to simulate chronic airway remodeling: OVA is a non- physiological allergen in humans, differing from clinical allergens	DNA methyltransferase inhibitors (5- azacytidine/5-azaC, decitabine)	Significantly decreases eosinophil infiltration in airway tissues; downregulates Th2-type cytokine (IL-4, IL-13) expression
OVA-induced chronic allergic asthma mouse model	Intraperitoneal sensitization with OVA followed by long-term repeated aerosol challenge	Recapitulates chronic inflammatory features and typical airway remodeling (e.g., smooth muscle hypertrophy, fibrosis), closely resembling clinical chronic asthma pathology	Relies on a single allergen (OVA), fails to mimic non-allergic asthma phenotypes; long construction cycle with relatively complex operations	miR-146a mimics; hypoxic hUCMSC-EVs (enriched with miR-146a-5p)	Inhibits NF-kB signaling pathway; significantly reduces inflammatory factors (IL-4, IL-13); alleviates airway inflammation and improves remodeling
IL-13 transgenic mouse model	Lung-specific overexpression of IL-13 in mice via transgenic technology	Directly simulates Th2-dominated asthma pathology; precisely focuses on the role of IL-13 in airway injury and inflammation, facilitating mechanistic research	Pathology driven by a single cytokine (IL- 13), ignoring synergistic effects of multiple cytokines and pathways, inconsistent with clinical complexity	miR-21 inhibitors (indirectly mentioned in source)	Restores IL-12p35 expression; corrects Th1/Th2 immune imbalance; reduces Th2- type inflammatory responses
HDM-induced chronic experimental allergic asthma mouse model	Repeated sensitization and aerosol challenge with house dust mite (HDM) extract	HDM is a common human asthma allergen, making the model clinically relevant; enables simulation of HDM-related chronic airway inflammation and remodeling	Fails to cover non-allergic asthma phenotypes; sensitivity to HDM varies among mouse strains, potentially affecting model reproducibility	LincR-PPP2R5C knockdown (genetic inhibition)	Activates PP2A/TGF-B1 signaling pathway; significantly reduces pro-inflammatory gene (e.g., IL-4, IL-13) expression; reverses EMT process, alleviating airway inflammation and remodeling
Primary human nasal epithelial cell (HNEC) ALI model	Isolation of primary HNECs; establishment of an air-liquid interface (ALI) culture system to mimic *in vivo* airway microenvironment	Mimics human airway epithelial physiological structure and barrier function; allows direct evaluation of barrier integrity (e.g., TEER); facilitates research on epithelial direct responses	*In vitro* model lacking complex immune microenvironment and intercellular interactions (e.g., epithelial-immune cell crosstalk); unable to fully simulate *in vivo* pathology	HDAC inhibitor (JNJ-26481585)	Significantly upregulates tight junction protein (Occludin, ZO-1) expression; increases transepithelial electrical resistance (TEER) by 50%–80%; repairs epithelial barrier function
TGF-β1-Stimulated human lung fibroblast (HLF-1) model	*In vitro* stimulation of human lung fibroblasts (HLF-1) with transforming growth factor-β1 (TGF-β1)	Focuses on core aspect of asthma airway remodeling (fibroblast activation and fibrosis); facilitates research on fibrosis- related mechanisms and interventions	Single-cell (fibroblast) model unable to simulate the complete “epithelial injury → inflammation → remodeling” cascade; lacks other cell types (e.g., epithelial, immune cells)	miR-146a mimics	Inhibits pro-inflammatory factor (TNF-α, IL- 1β) expression; reduces TGF-B1-induced fibroblast activation and collagen synthesis; alleviates fibrotic tendency

#### 5.1.4 Technical challenges in translational research

Technical limitations constrain translational application of epigenetic findings. Invasive procedures such as bronchoscopy, which are required for airway epithelial cell collection, hinder large-scale studies. Epigenetic data stability is also compromised by variations in sample processing and storage, introducing potential biases (57). Furthermore, optimizing therapeutic dosages and minimizing off-target effects for epigenetic-based therapies present unresolved challenges.

### 5.2 Future research priorities

#### 5.2.1 Technological improvements and standardization

Advanced preclinical models, particularly humanized mouse systems, are urgently needed to more accurately recapitulate the pathogenic mechanisms of pediatric asthma. Standardization of non-invasive sampling methods (e.g., nasal swabs) and epigenetic detection protocols is critical to improving data reliability. For example, in ALOX12 methylation analysis, the consistency of results between nasal swab and peripheral blood samples has not been validated in multicenter studies, and unified cut-off values are still lacking. Thus, establishing standardized control criteria for different sample types should be prioritized. Rigorous preclinical studies are essential to optimize epigenetic drug dosing and delivery methods and reduce off-target effects.

#### 5.2.2 Multi-omics integration: mechanisms, infection-associated methylome changes, and endotype specificity

A multi-omics approach–integrating epigenomics with genomics, transcriptomics, and proteomics–is critical for unraveling the multifactorial pathogenesis of asthma. Specifically, this cross-omics integration can decipher the crosstalk between epigenetic modifications and immune responses, cytokine networks, and airway remodeling, thereby facilitating the discovery of novel biomarkers (24). This value is particularly evident in studies linking infection to methylome alterations and endotype-specific barrier dysfunction: Rhinovirus infection, a major asthma exacerbator, induces coordinated DNA methylation and mRNA changes in asthmatic toddlers: 93 differentially methylated CpGs (e.g., CCL24 hypomethylation) correlate with 2.1-fold upregulation of inflammatory transcripts, directly linking viral exposure to epithelial inflammation (30, 58). Similarly, SARS-CoV-2 nsp1 mediates H3K9me2 histone modification at tight junction loci (CLDN5/OCLN), repressing their expression and impairing barrier integrity–findings only achievable via cross-omics analysis (33). In T2-high asthma, nasal epithelial epigenome-wide studies further confirm POSTN/NTRK2 hypomethylation correlates with upregulated mRNA, validating epigenetic-transcriptional crosstalk in endotype-specific barrier defects (59).

#### 5.2.3 Exploring the epigenetic mechanisms and temporal dynamics of environmental stressors

The dynamic interplay between environmental stressors and epigenetic modifications warrants focused investigation. Future studies should delineate how chronic exposure to pollutants or allergens induces epigenetic changes over time, shaping immune dysfunction and epithelial repair. Future research must prioritize early-life exposures–specifically, synchronously monitoring pollutant (e.g., PM2.5) or allergen exposure and methylome dynamics of barrier genes (e.g., FLG, CLDN) in birth cohorts–to clarify the “early exposure window-epigenetic change-later asthma risk” causal chain, addressing the current research gap of insufficient cohorts focusing on participants < 3 years old.

#### 5.2.4 Developing treatment strategies based on epigenetic characteristics

Translating preclinical findings into clinical therapies is imperative. Targeted modulation of DNA methylation (e.g., at FLG, CLDN loci), histone modifications, and ncRNAs offers a testable mechanistic hypothesis for restoring barrier function and mitigating inflammation, but its clinical relevance awaits formal evaluation in pediatric cohorts. Preclinical evidence supports this potential: the HDAC inhibitor JNJ-26481585 increases the expression of tight junction proteins (occludin and ZO-1) and enhances airway barrier integrity in air-liquid interface (ALI) cultures of primary human nasal epithelial cells (HNECs), with occludin expression and transepithelial electrical resistance (TEER) increased by >50% and >80%, respectively (24, 50). Combining epigenetic therapies with existing immunomodulators (e.g., anti-IL-4/IL-13 antibodies) provides a mechanistic rationale for future phase II pediatric trials; clinical benefit remains to be demonstrated (26, 55, 60). Notably, no epigenetic drugs for pediatric asthma have entered Phase III clinical trials, highlighting the need to advance translation of preclinically effective agents to pediatric populations.

#### 5.2.5 Large cohort validation and clinical translational challenges

Large pediatric cohorts are essential to advance epigenetic biomarkers (e.g., ALOX12 hypomethylation) toward clinical use, but key gaps remain. The INMA project (Menorca: *n* = 122; Sabadell: *n* = 236) cross-validated ALOX12 hypomethylation as a persistent wheezing predictor (OR = 1.13/1.16 per 1% methylation decrease), and supplementing epigenetic data from pediatric populations in different regions (e.g., Chinese children) could further enhance the population applicability of this biomarker, but lacks discussion on methylation assay standardization–critical for consistent clinical testing (39). Additionally, as noted in Section “5.2.3 Exploring the epigenetic mechanisms and temporal dynamics of environmental stressors,” this includes the research gap of insufficient cohorts focusing on participants < 3 years old–a limitation equally prominent in large-cohort validation: a Pregnancy And Childhood Epigenetics Consortium meta-analysis (6 cohorts, *n* = 2695) found only 12%–15% of participants were <3 years old, limiting validation of early-life predictors (when barrier dysfunction originates) (61). Future work must prioritize longitudinal birth cohorts to address population variability, standardize detection methods (e.g., nasal swab vs. blood), and resolve the practical challenges of integrating epigenetic biomarkers into routine pediatric asthma diagnosis and management (e.g., aligning with existing diagnostic workflows).

## 6 Conclusion

Epigenetic discoveries unlock novel mechanistic and therapeutic avenues in pediatric asthma, heralding a new era of precision care. While challenges in clinical translation persist, advancements in biomarker discovery, multi-omics integration, and targeted epigenetic therapies hold transformative potential for precision medicine. Collaborative efforts to address current limitations will be critical to achieving individualized asthma management with improved efficacy and safety.
